# Supplementary material for: Synthesis of Network Biobased Aliphatic Polyesters Exhibiting Better Tensile Properties than the Linear Polymers by ADMET Polymerization in the Presence of Glycerol Tris(undec-10-enoate)
Source: Polymers (Basel). 2024 Feb 7;16(4):468. doi: 10.3390/polym16040468 (PMC10891964; doi:10.3390/polym16040468)
Supplement: Supplementary file 1 [file polymers-16-00468-s001.zip › polymers-2826608-supplementary.pdf]

Supplementary Materials

for

Synthesis of Soluble Network Biobased Aliphatic Polyesters Exhibiting Better Tensile Properties than the Linear Polymers by ADMET Polymerization of Bis(undec-10-enoate) with Isosorbide in the Presence of Tris(undec-10-enoate) with Glycerol

Lance O'Hari P. Go [1], Mohamed Mehawed Abdellatif [1], Ryoji Makino [1], Daisuke Shimoyama [1], Seiji Higashi [2], Hiroshi Hirano [2] and Kotohiro Nomura [1,*]

[1] Department of Chemistry, Tokyo Metropolitan University, 1-1 Minami Osawa, Hachioji, Tokyo 192-0397, Japan
[2] Osaka Research Institute of Industrial Science and Technology (ORIST), 1-6-50, Morinomiya, Joto-ku, Osaka 536-8553, Japan
* Correspondence: ktnomura@tmu.ac.jp

Contents

		page
1.	Selected NMR spectra of the prepared monomer, cross linker, and polymers.	S2-S7
2.	Selected GPC traces of the prepared polymers.	S8,9
3.	Additional results for the mechanical properties of the prepared polymers.	S10,11
4.	Depolymerization of the prepared polyesters by catalytic transesterification using $CpTiCl_3$.	S12-14
5.	Thermal properties of the prepared polyesters.	S15

1. Selected NMR spectra of the prepared monomer, cross linker, and polymers.

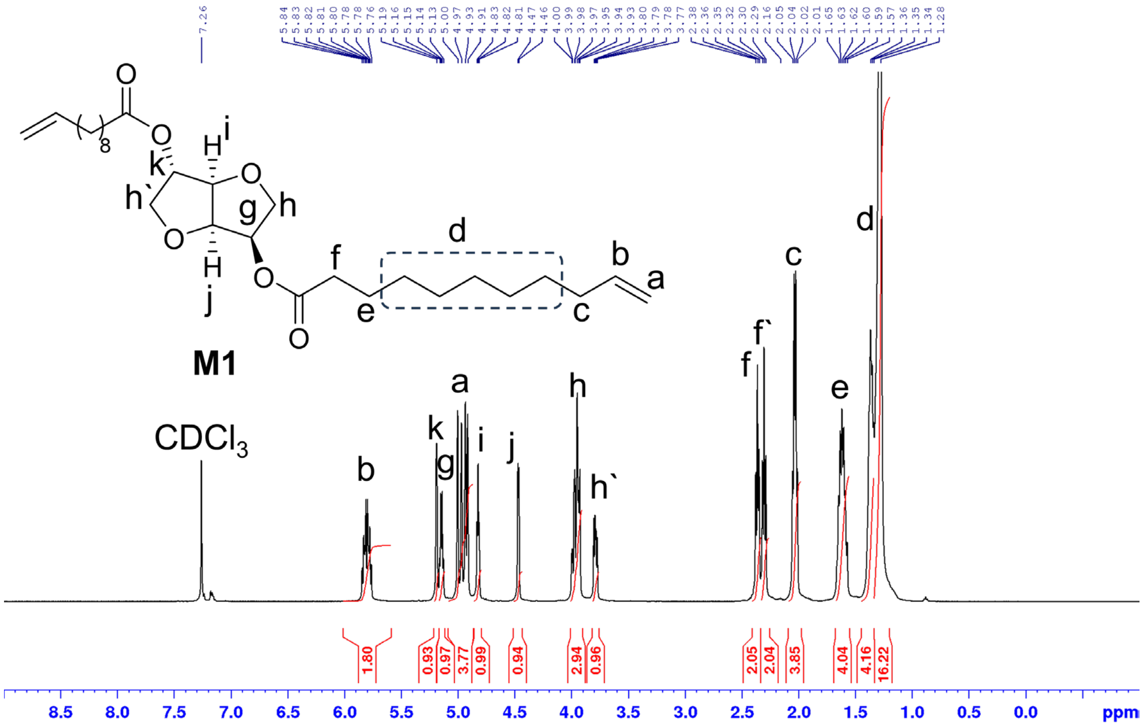

Figure S1. ^{1}H NMR spectrum (in $CDCl_3$ at 25 ˚C) for monomer (**M1**).

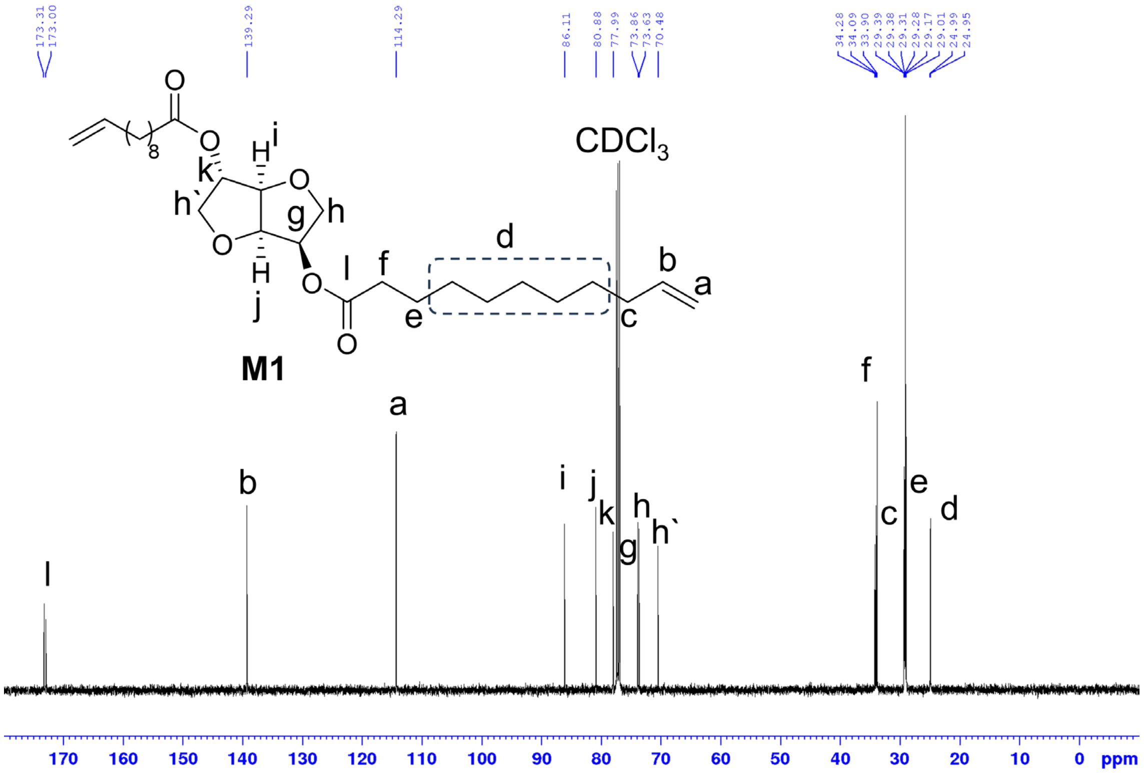

Figure S2. ^{13}C NMR spectrum (in $CDCl_3$ at 25 ˚C) for **M1**.

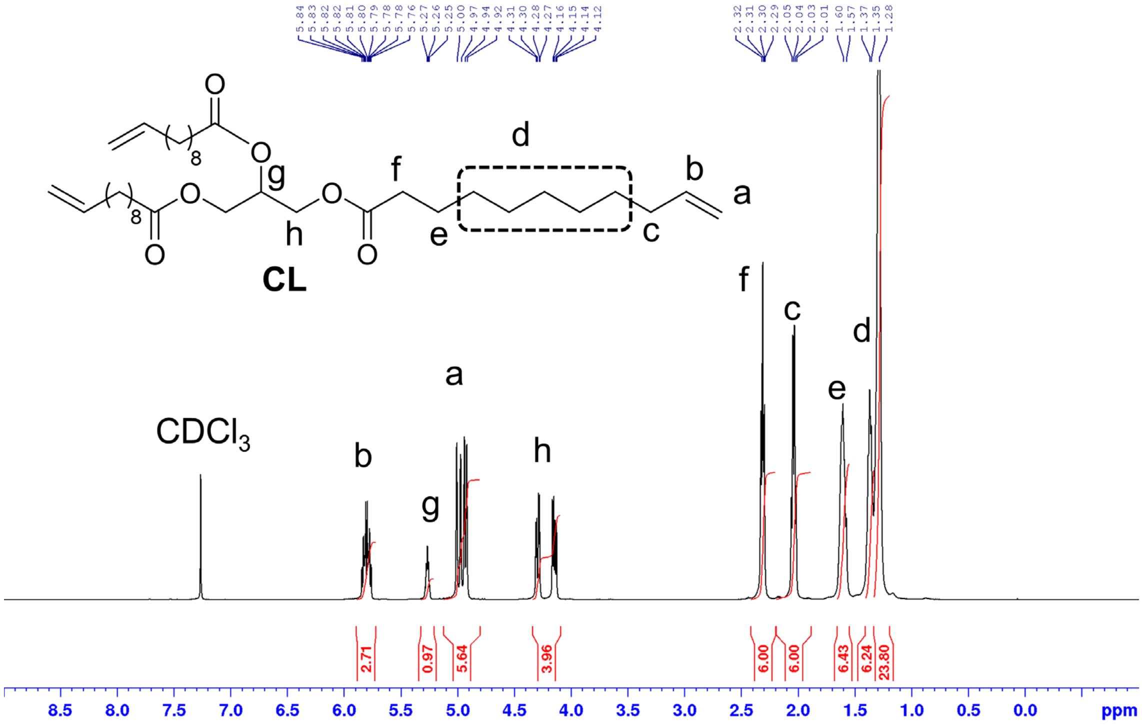

Figure S3. ^{1}H NMR spectrum (in $CDCl_3$ at 25 ˚C) for cross linker (**CL**).

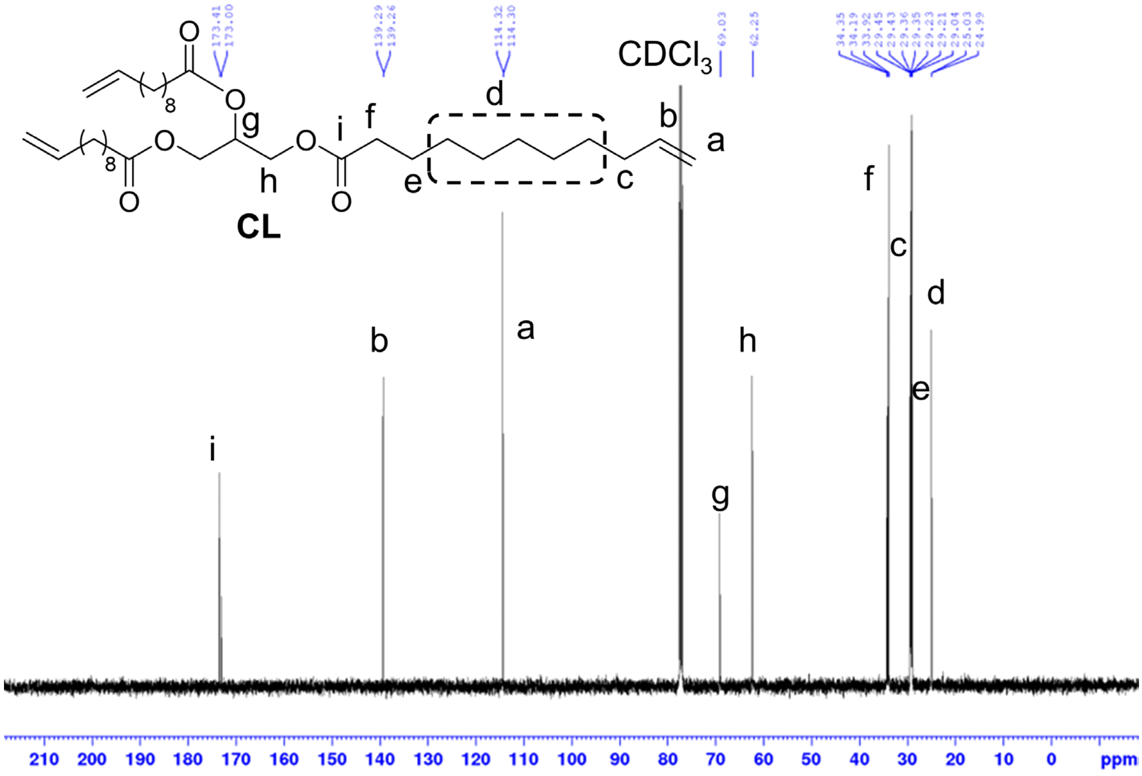

Figure S4. ^{13}C NMR spectrum (in $CDCl_3$ at 25 ˚C) for cross linker (**CL**).

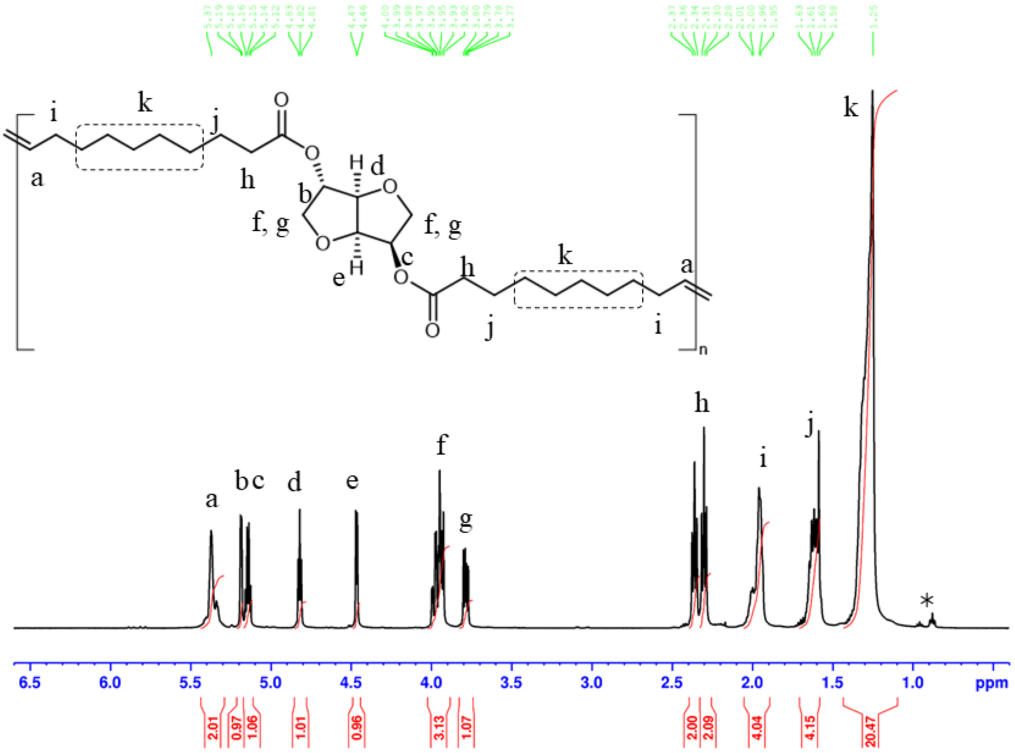

Figure S5. ^{1}H NMR spectrum (in $CDCl_3$ at 25 ˚C) for ADMET polyester (**P1**).

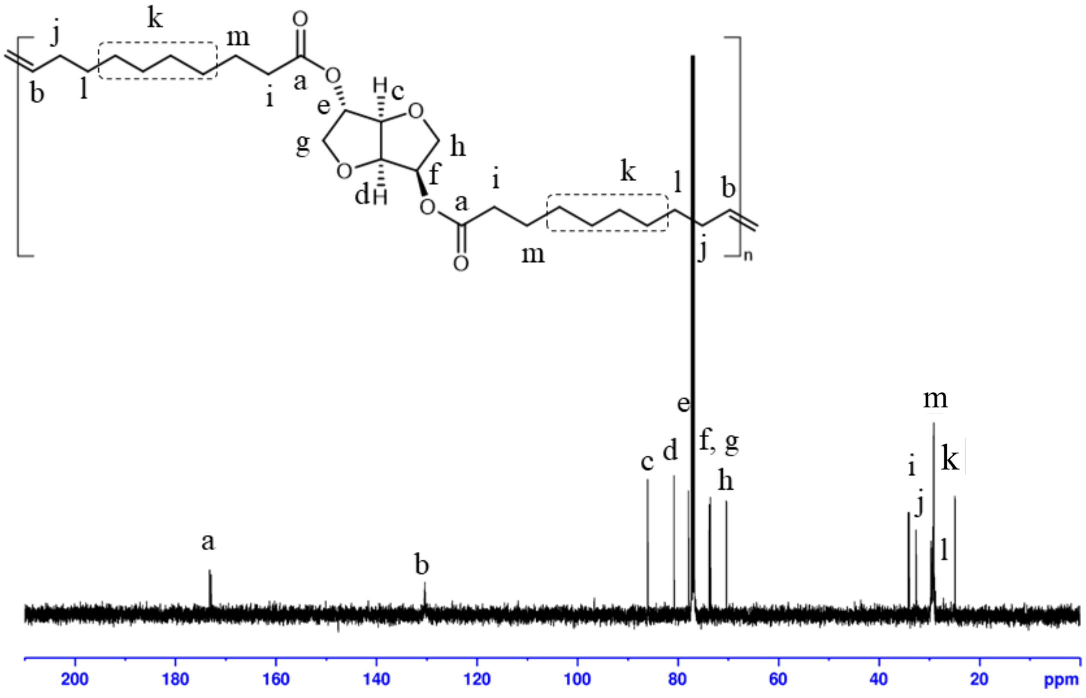

Figure S6. ^{13}C NMR spectrum (in $CDCl_3$ at 25 ˚C) for ADMET polyester (**P1**).

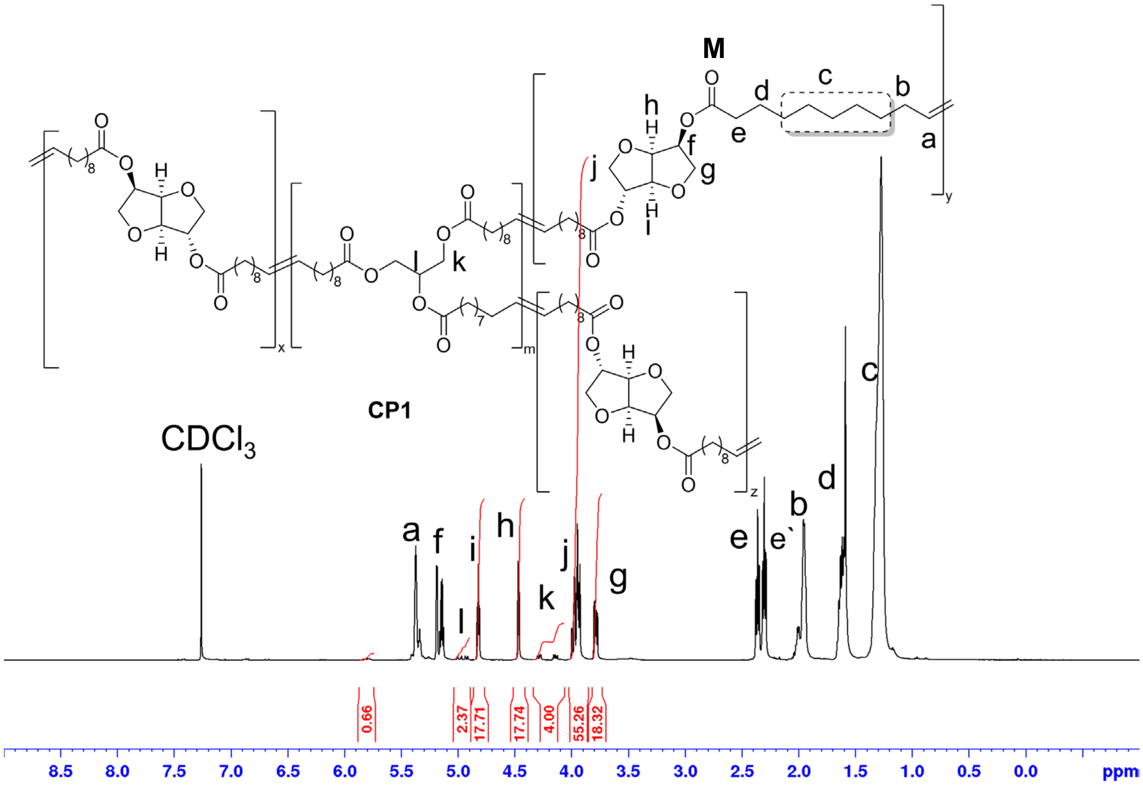

Figure S7. ^{1}H NMR spectrum (in $CDCl_3$ at 25 ˚C) for cross linked ADMET polyester (**CP1**).

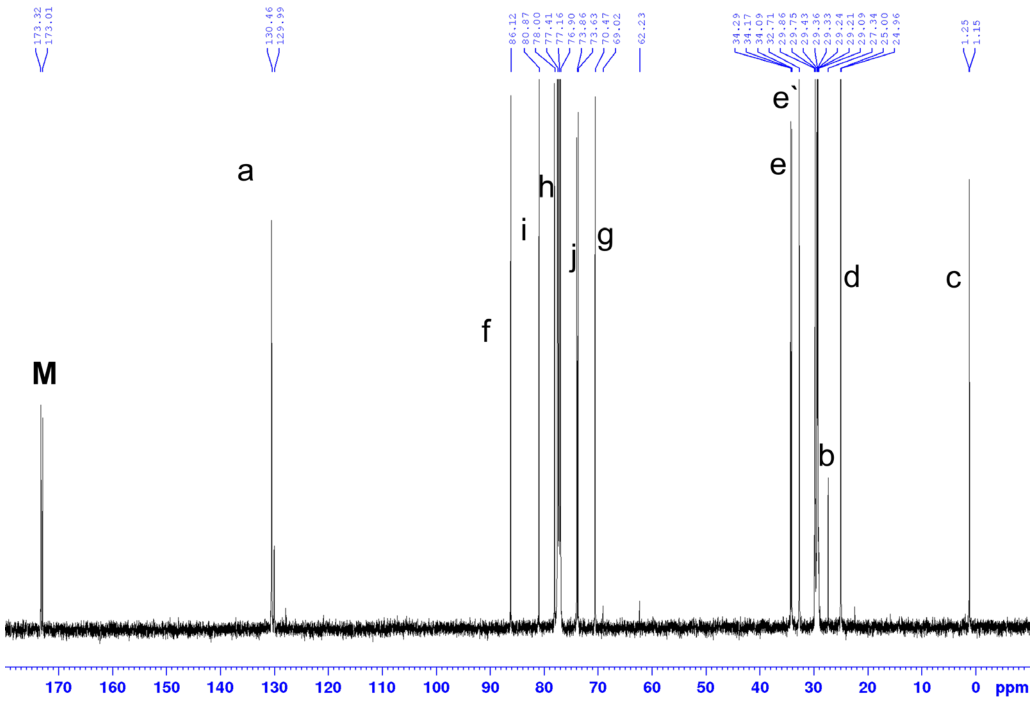

Figure S8. ^{13}C NMR spectrum (in $CDCl_3$ at 25 ˚C) for cross linked ADMET polyester (**CP1**).

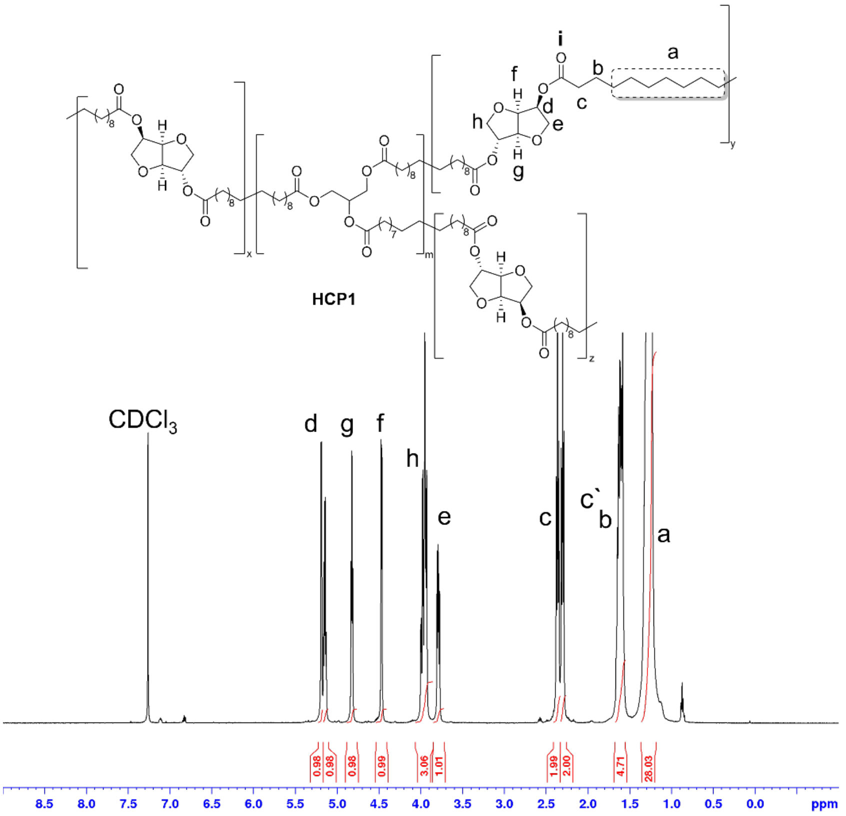

Figure S9. ^{1}H NMR spectrum (in $CDCl_3$ at 25 ˚C) for hydrogenated ADMET polyester (**HCP1**, Table 2- run 42) with 1.0 mol% of the cross linker (**CL**).

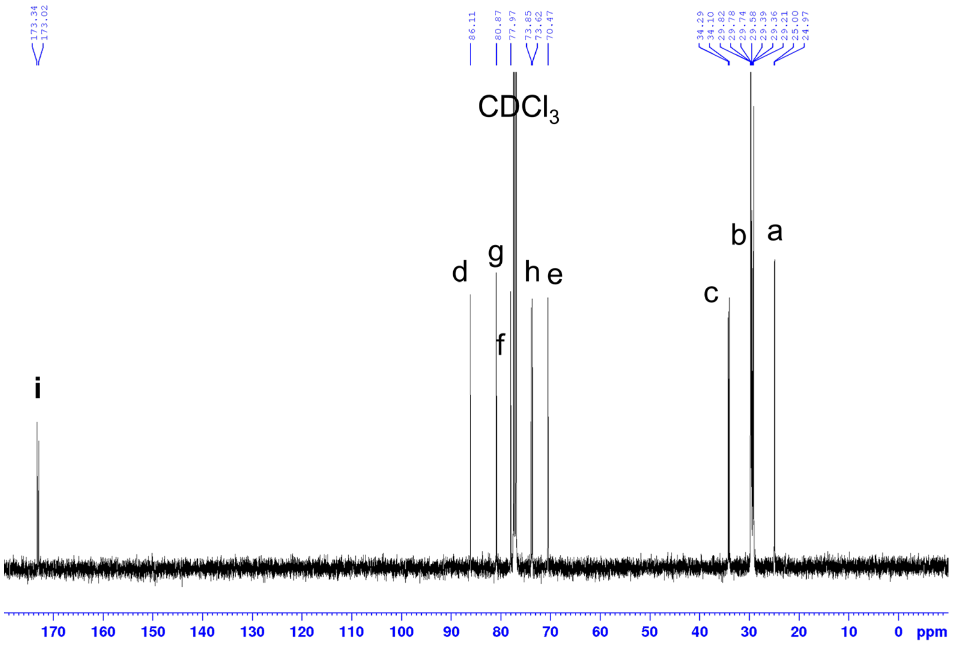

Figure S10. ^{13}C NMR spectrum (in $CDCl_3$ at 25 ˚C) for hydrogenated ADMET polyester (**HCP1**, Table 2- run 42) with 1.0 mol% of the cross linker (**CL**).

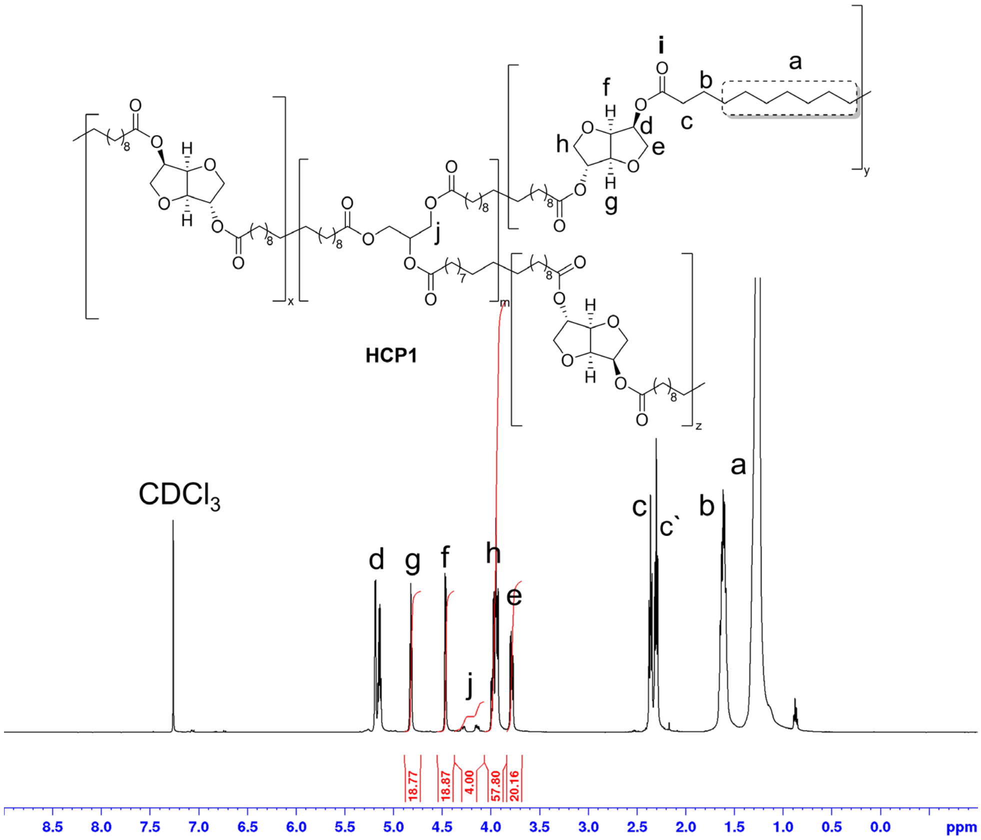

Figure S11. ^{1}H NMR spectrum (in $CDCl_3$ at 25 ˚C) for hydrogenated cross linked ADMET polyester (**HCP1**, Table 2- run 41) with 2.5 mol% of the cross linker (**CL**).

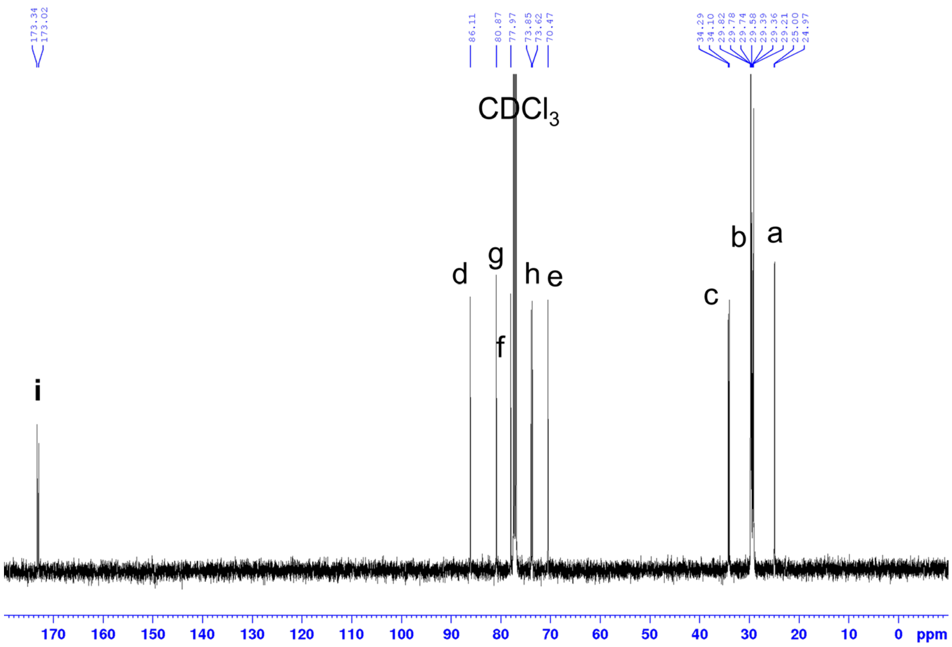

Figure S12. ^{13}C NMR spectrum (in $CDCl_3$ at 25 °C) for hydrogenated cross linked ADMET polyester (**HCP1**, Table 2- run 41) with 2.5 mol% of the cross linker (**CL**).

2. Selected GPC traces of the prepared polymers.

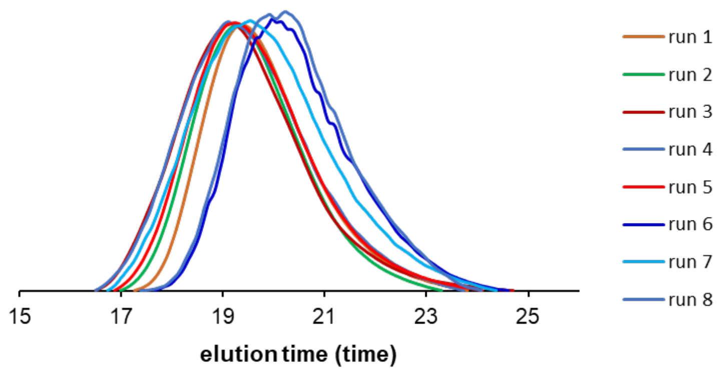

Figure S13. GPC traces for polyesters (**P1**) and cross linked polyesters (**CP1s)** with 0.5 and 1.0 mol% cross linker using a one step approach. (Table 1, runs 1-8)

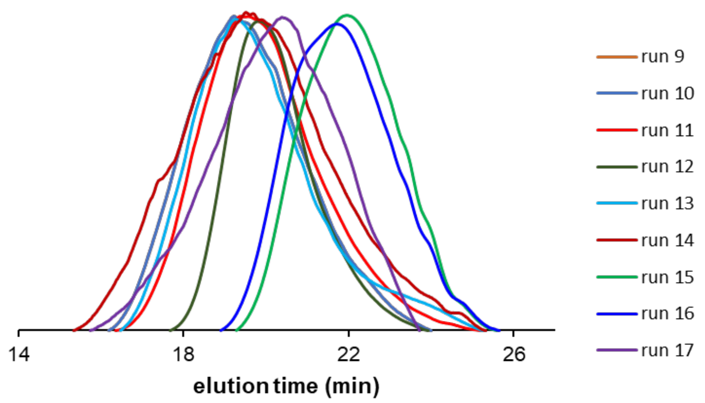

Figure S14. GPC traces for cross linked polyesters (**CP1s)** with 2.5 and 5.0 mol% cross linker using a one step approach. (Table 1, runs 9-16)

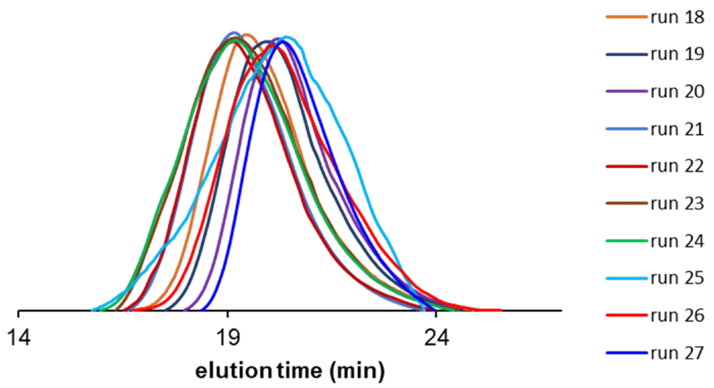

Figure S15. GPC traces for cross linked polyesters (**CP1s)** with 2.5 and 5.0 mol% cross linker using a two steps approach. (Table 1, runs 18-27)

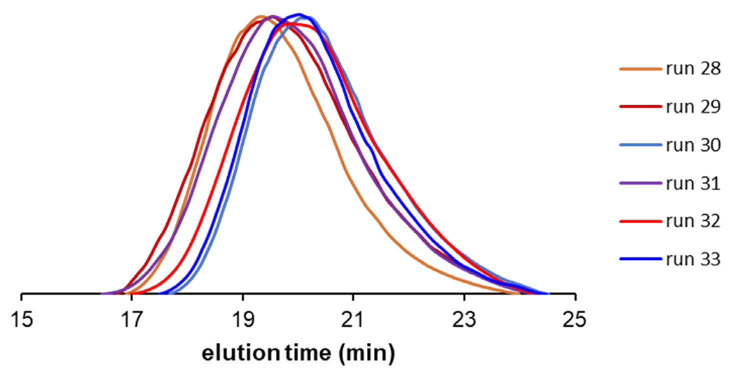

Figure S16. GPC traces for cross linked polyesters (**CP1s)** with 2.5 and 5.0 mol% cross linker using a two steps approach. (Table 1, runs 28-33)

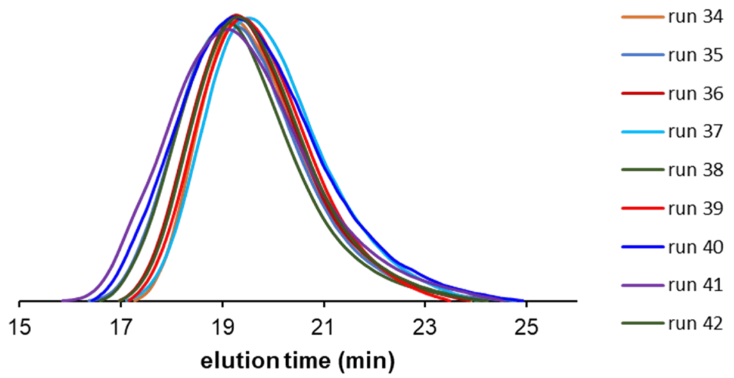

Figure S17. GPC traces for hydrogenated polyester (**HP1**) and hydrogenated cross linked polyesters (**HCP1s)** with 1.0 and 2.5 mol% cross linker using one step and two steps approaches. (Table 1, runs 34-42)

3. Additional results for the mechanical properties of the prepared polymers.

Table S1. Summary of the tensile properties of unsaturated network polyesters at a speed of 10 mm/min (23 ºC, humidity 50±10 %).[a]

sample	CL / mol% [approach]	M_n[b] ×10^{-3}	M_w/M_n[b]	tensile strength / MPa	elongation at break / %	toughness / MJ/m^3	yield strength / MPa
**P1**[c]	0	39.6	1.89	17.3 (±2.2)	506 (±44)	49 (±7)	4.8 (±0.4)
**CP1** (run 21)	1.0 [2 step]	36.6	2.79	24.6 (±1.1)	798 (±47)	95 (±8)	4.6 (±0.1)
**CP1** (run 22)	1.0 [2 step]	35.8	3.00	21.3 (±2.0)	816 (±48)	88 (±9)	3.7 (±0.2)
**CP1** (run 4)	1.0 [1 step]	34.8	3.11	18.6 (±0.5)	807 (±5)	74 (±3)	3.9 (±0.2)
**CP1** (run 1)	0	30.3	2.11	15.4 (±1.2)	444 (±28)	41 (±4)	4.3 (±0.2)
**CP1** (run 28)	1.0 [2 step]	31.2	2.54	17.2 (±0.7)	707 (±21)	65 (±4)	4.6 (±0.1)
**CP1** (run 5)	1.0 [1 step]	32.2	2.64	18.4 (±0.7)	710 (±66)	68 (±9)	4.5 (±0.3)
**CP1** (run 23)	2.5 [2 step]	31.1	4.13	19.7 (±1.5)	704 (±59)	70 (±9)	4.2 (±0.1)
**CP1** (run 10)	2.5 [1 step]	29.6	4.14	17.8 (±1.3)	696 (±19)	67 (±4)	4.4 (±0.1)
**CP1** (run 24)	2.5 [2 step]	32.0	4.48	19.8 (±1.4)	578 (±101)	61 (±15)	4.3 (±0.1)

[a]Stress/strain experiments were conducted using Shimadzu Universal Testing Instruments (Autograph AGS-10kNX, max load cell capacity of 500 N). The test specimens had the following dimensions: a gauge length of 1.0 mm; a width of 1.0 cm; a length of 2.5 cm; and a thickness of 0.1 mm. [b]GPC data in THF *vs* polystyrene standards. [c]Cited from reference **1**.

Table S2. Summary of the tensile properties of hydrogenated linear and network polyesters at a speed of 10 mm/min (23 ºC, humidity 50±10 %).[a]

sample	CL / mol% [approach]	M_n[b] ×10^{-3}	M_w/M_n[b]	tensile strength / MPa	elongation at break / %	toughness / MJ/m^3	yield strength / MPa
**HP1**[c]	0	40.9	2.41	33.7 (±2.2)	413 (±13)	74 (±7)	7.3 (±0.8)
**HCP1** (run 38)	1.0 [2 step]	37.3	2.92	36.9 (±3.8)	555 (±21)	126 (±13)	9.5 (±0.4)
**HCP1** (run 39)	1.0 [2 step]	35.3	2.98	32.5 (±2.4)	515 (±47)	101 (±17)	8.2 (±0.8)
**HCP1** (run 35)	1.0 [1 step]	34.8	3.10	29.1 (±4.3)	453 (±74)	82 (±24)	8.5 (±0.2)
**HCP1** (run 34)	0	30.7	2.22	20.8 (±1.3)	282 (±14)	37 (±4)	8.1 (±0.2)
**HCP1** (run 42)	1.0 [2 step]	31.4	2.46	35.4 (±0.6)	572 (±1)	125 (±1)	9.6 (±0.2)
**HCP1** (run 36)	1.0 [1 step]	32.6	2.47	34.7 (±0.6)	537 (±7)	116 (±4)	9.6 (±0.2)
**HCP1** (run 40)	2.5 [2 step]	30.5	3.91	31.9 (±1.6)	457 (±65)	94 (±18)	9.9 (±0.1)
**HCP1** (run 37)	2.5 [1 step]	28.3	4.05	33.0 (±0.6)	463 (±58)	99 (±16)	10.5 (±0.1)
**HCP1** (run 41)	2.5 [2 step]	32.2	4.39	23.7 (±7.3)	228 (±55)	37 (±16)	10.3 (±0.5)

[a]Stress/strain experiments were conducted using a compact high-performance tensile tester by Acroedge Co., Ltd. (Stency model OZ918, max load cell capacity of 50 N). The test specimens had the following dimensions: a gauge length of 1.0 mm; a width of 1.0 cm; a length of 2.5 cm; and a thickness of 0.1 mm. [b]GPC data in THF vs polystyrene standards. [c]Cited from reference **1**.

4. Depolymerization of saturated network polyesters by catalytic transesterification.

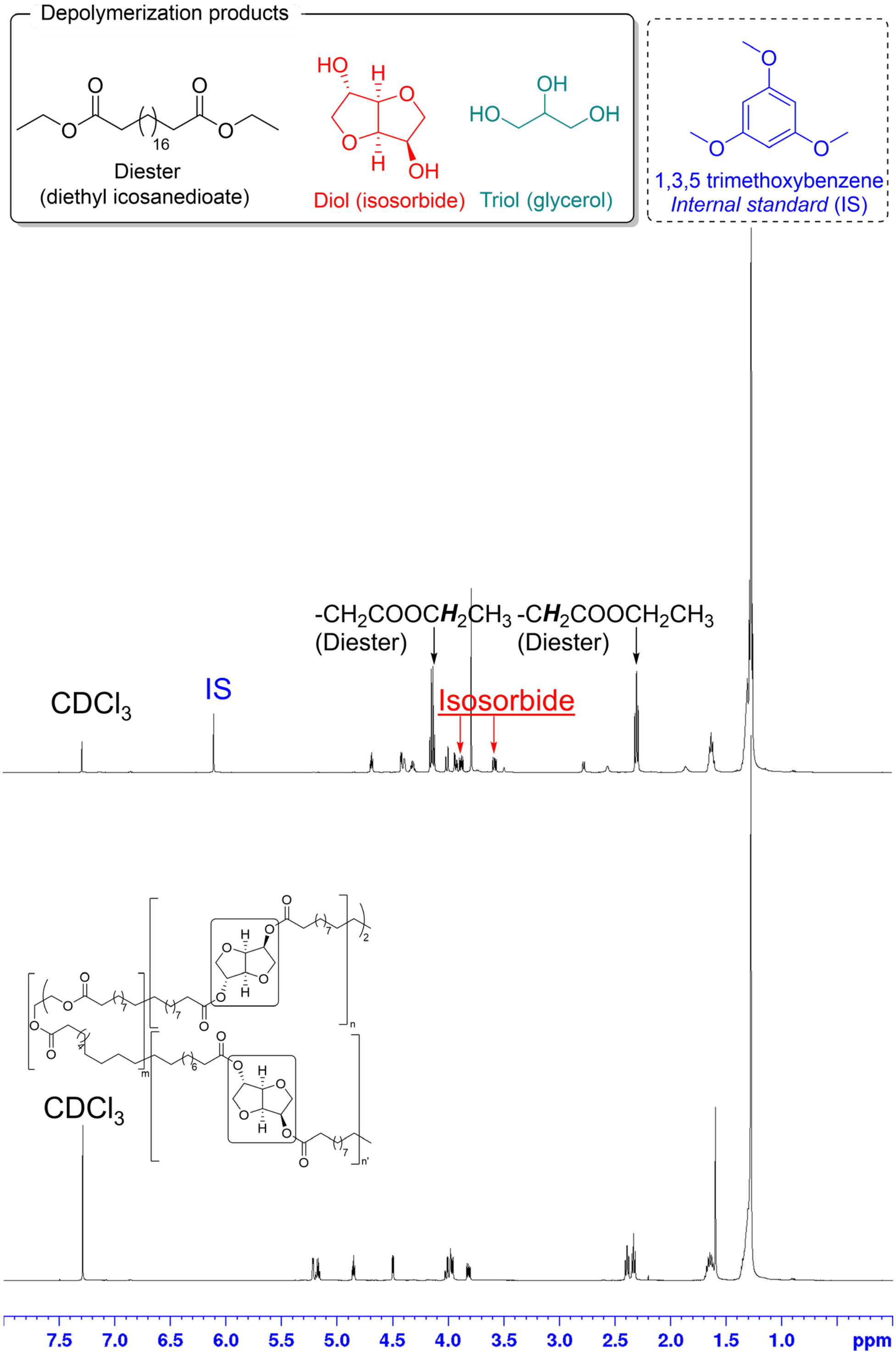

Figure S18. ^{1}H NMR spectrum (in $CDCl_3$ at 25 °C) of (top) depolymerized network polyester (**HCP1**, Table 2, run 41) and (bottom) network polyester (**HCP1**, Table 2, run 41). IS: Internal standard (1, 3, 5-trimethoxybenzene).

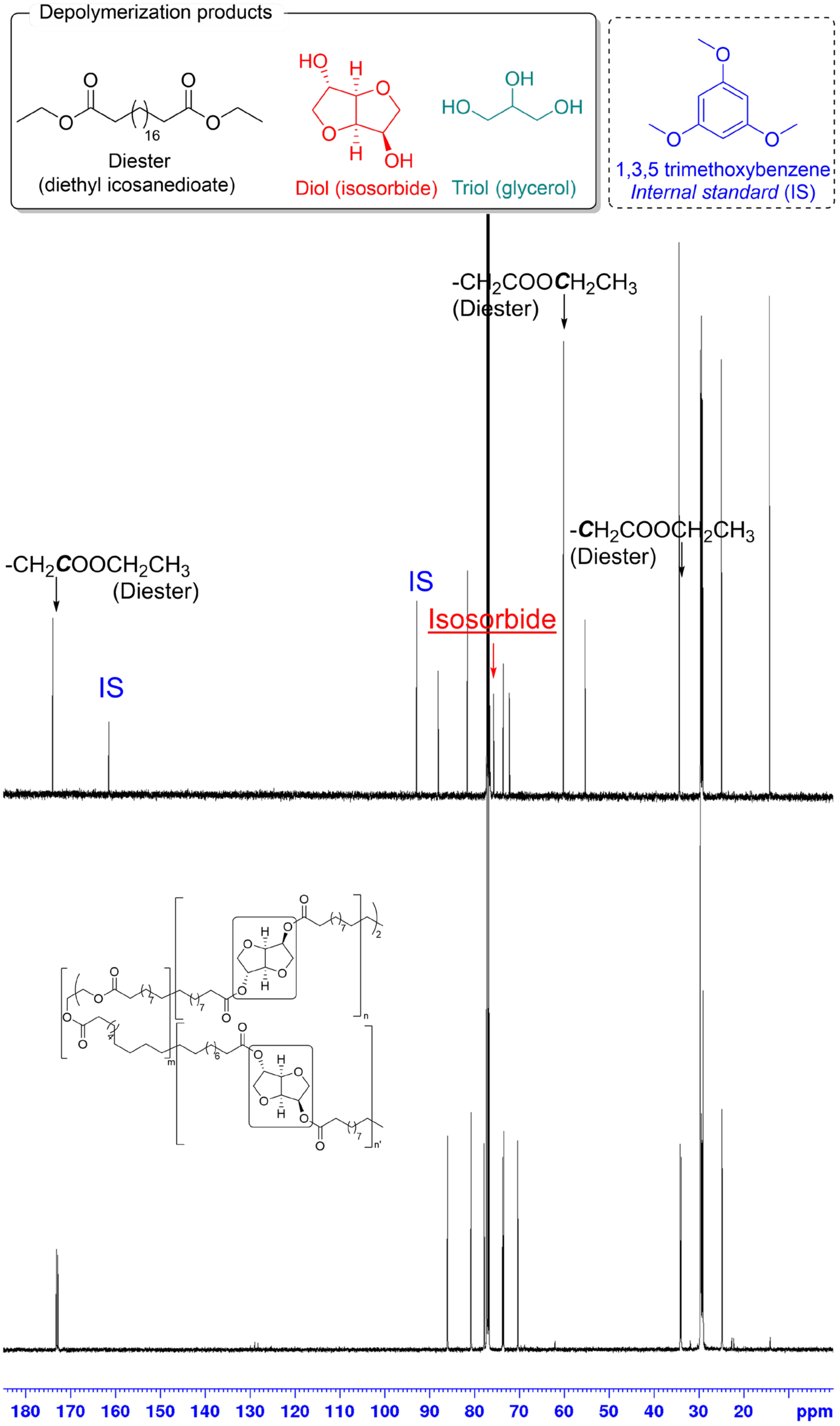

Figure S19. ^{13}C NMR spectrum (in $CDCl_3$ at 25 °C) of (top) depolymerized network polyester (**HCP1**, Table 2, run 41) and (bottom) network polyester (**HCP1**, Table 2, run 41). IS: Internal standard (1, 3, 5-trimethoxybenzene).

Table S3. Depolymerization of hydrogenated network polyesters by catalytic transesterification in ethanol using $CpTiCl_3$.[a]

sample	CL / mol%	M_n[b] / g.mol^{-1}	M_w/M_n[b]	conv.[c] / %	yield isosorbide[d] / %	yield diester[e] / %
**HPECL** (run 38)	1.0	37,300	2.92	>99	>99	96
**HPECL** (run 41)	2.5	32,200	4.39	>99	>99	94

[a]Conditions: network polyester 100 mg, ethanol 2.0 ml, $CpTiCl_3$ 1.0 mol%, 150 °C, 24 h. [b]GPC data in THF vs. polystyrene standards. [c]Estimated by ^{13}C NMR. [d]From GC yield *vs.* internal standard. [e]From ^{1}H NMR yield *vs.* internal standard.

5. Thermal properties of the prepared polyesters.

Table S4 . Thermal properties of the prepared linear and network polyesters.

before hydrogenation				after hydrogenation			
sample no.[1]	M_n[3]$\times 10^{-4}$	M_w/M_n[3]	T_m[4]/ °C	sample no.[2]	M_n[3]$\times 10^{-4}$	M_w/M_n[3]	T_m[4]/ °C
run 1	3.03	2.11	32.7	run 34	3.07	2.22	72.8
run 28	3.20	4.48	31.3	run 42	3.22	4.39	72.7
run 24	3.12	2.54	30.8	run 41	3.14	2.46	73.0

[1] Run number in Table 1. [2] Run number in Table 2. [3] GPC data in THF *vs.* polystyrene standards. [4] By DSC thermograms.

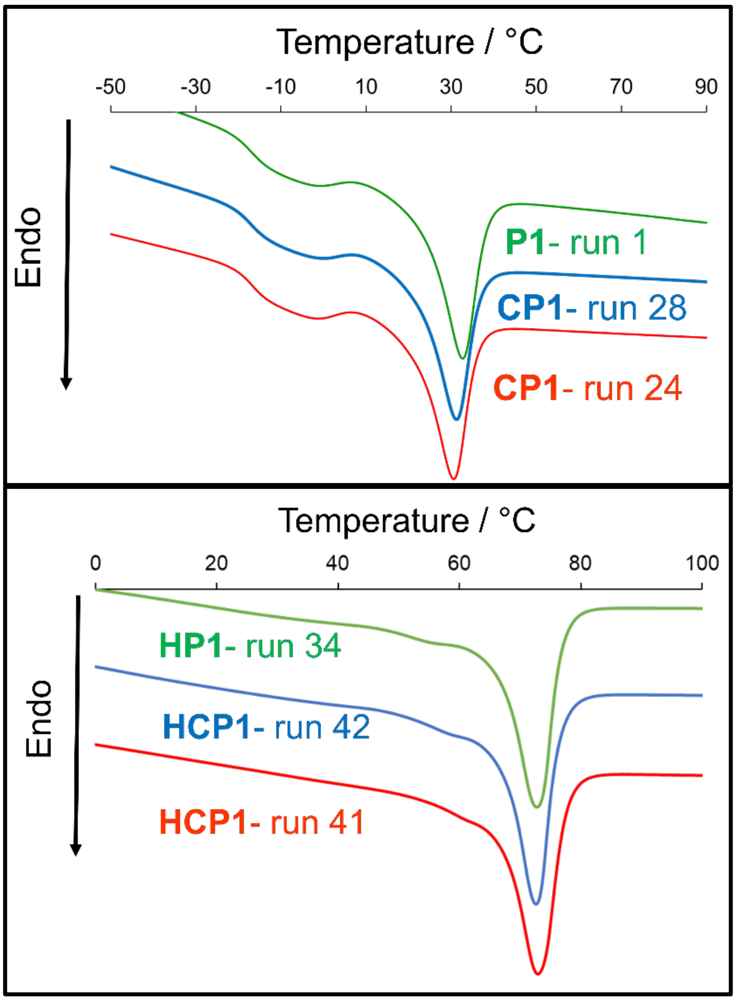

Figure S20. DSC thermograms of the resultant linear and network polyesters (top) ADMET polymers (bottom) after hydrogenation.

References

1. Kojima, M.; Wang, X.; Go, L. O. P.; Makino, R.; Matsumoto, Y.; Shimoyama, D.; Abdellatif, M. M.; Kadota, J.; Higashi, S.; Hirano, H.; Nomura, K. Synthesis of high molecular weight biobased aliphatic polyesters exhibiting tensile properties beyond polyethylene. *ACS Macro Lett.* **2023**, *12*, 1403–1408.
